# Brain Processing of Biologically Relevant Odors in the Awake Rat, as Revealed by Manganese-Enhanced MRI

**DOI:** 10.1371/journal.pone.0048491

**Published:** 2012-10-31

**Authors:** Benoist Lehallier, Olivier Rampin, Audrey Saint-Albin, Nathalie Jérôme, Christian Ouali, Yves Maurin, Jean-Marie Bonny

**Affiliations:** 1 UR370 QuaPA, INRA, Saint-Genès-Champanelle, France; 2 UR1197 NOeMI, INRA, Jouy-en-Josas, France; Duke University, United States of America

## Abstract

**Background:**

So far, an overall view of olfactory structures activated by natural biologically relevant odors in the awake rat is not available. Manganese-enhanced MRI (MEMRI) is appropriate for this purpose. While MEMRI has been used for anatomical labeling of olfactory pathways, functional imaging analyses have not yet been performed beyond the olfactory bulb. Here, we have used MEMRI for functional imaging of rat central olfactory structures and for comparing activation maps obtained with odors conveying different biological messages.

**Methodology/Principal Findings:**

Odors of male fox feces and of chocolate flavored cereals were used to stimulate conscious rats previously treated by intranasal instillation of manganese (Mn). MEMRI activation maps showed Mn enhancement all along the primary olfactory cortex. Mn enhancement elicited by male fox feces odor and to a lesser extent that elicited by chocolate odor, differed from that elicited by deodorized air. This result was partly confirmed by c-Fos immunohistochemistry in the piriform cortex.

**Conclusion/Significance:**

By providing an overall image of brain structures activated in awake rats by odorous stimulation, and by showing that Mn enhancement is differently sensitive to different stimulating odors, the present results demonstrate the interest of MEMRI for functional studies of olfaction in the primary olfactory cortex of laboratory small animals, under conditions close to natural perception. Finally, the factors that may cause the variability of the MEMRI signal in response to different odor are discussed.

## Introduction

In small animal models, an overall picture of the activation of the primary olfactory cortex following stimulation by natural biologically relevant odors has not yet been produced. This is likely due to the inadequacy of most functional imaging techniques to provide such global views. *Ex vivo* techniques, such as 2-deoxyglucose consumption [1]–[3] and c-Fos immunodetection [4]–[8] require processing of serial brain sections, which practically limits their application to restricted, previously identified regions and prevents from using them as exploratory approaches. *In vivo* imaging techniques, such as 2-photon calcium imaging and positron emission tomography (PET) also have limitations. *In vivo* calcium imaging [9]–[11] is limited to restricted brain surface regions and PET scanning, which produces three-dimensional images of the whole brain, has a low spatial resolution that has largely limited its application to human studies [12]–[15]. Functional magnetic resonance imaging (fMRI) may seem more appropriate as it can now provide three-dimensional images of the whole rat brain with a voxel size in the 50–100 µm range [16]. However, blood oxygen level dependent (BOLD) contrast, used in most fMRI studies [17]–[19] requires immobility of the subject in order to perform image acquisition during stimulus application. When applied to rats, these should be either habituated to restraint or anaesthetized to ensure immobility. Unfortunately, anesthesia decreases brain baseline activity [20], [21] and blocks the brain ability to integrate information [22]. Habituation to restraint allows BOLD fMRI to be performed on conscious animals [23] and has been used in rats for olfaction studies [24]–[28]. However, besides being practically challenging, this approach may be hampered by motion artifacts and by the risk of substituting the adverse effects of anesthesia with those of restraint stress [29].

An alternative to BOLD fMRI is manganese-enhanced MRI (MEMRI), which allows avoiding both anesthesia and restraint during stimulation [30]. MEMRI relies upon three features: i) Mn has paramagnetic properties that make it detectable by MRI, ii) Mn is a calcium analogue and, as such, is taken up by activated neurons [31] and migrates along neuronal processes and finally iii) Mn is slowly eliminated by neurons [32]. In Mn injected animals, the combination of these three properties allows stimulations to be performed on conscious and free-moving animals and images to be acquired *a posteriori* under anesthesia. Image contrast depends on neuronal Mn concentration, which partly depends upon Mn bioavailability, hence upon its mode of administration. In the present study, targeting olfactory regions, we administered Mn into the nostrils, a method previously used in mice [33], [34] and in starlings [35]. However, MEMRI suffers two major drawbacks. First, because of Mn neurotoxicity [36], the intranasally administered dose must be minimized, yet remain high enough to ensure bioavailability in distal olfactory cortex regions. We have determined an appropriate dose that satisfies these two criteria [37]. Secondly, because of Mn persistence, an animal cannot undergo alternate resting and stimulation phases, which would allow calculating an individual activation map. In other words, an animal cannot be its own control. Stimulated and control animals compose different groups. In order to obtain activation maps, images from stimulated and control groups have to be normalized before being compared. For this purpose, we have designed and validated an algorithm for spatial and intensity normalization of MEMRI data sets [38].

In the present study, we have taken advantage of these two methodological improvements (optimized Mn dose and image normalization) to compare Mn enhanced images of the olfactory system (i.e. the olfactory bulb and beyond) following stimulation of conscious Brown-Norway rats with odors conveying different biologically relevant messages. The odors were those of male fox feces and of chocolate-flavored cereals. Fox is a natural predator of the rat and its feces give off an odor that triggers an innate fear reaction [39], while chocolate is a food reward [40]. We show here that these odors differently activate central olfactory pathways, as shown both by MEMRI and c-Fos immunodetection in the piriform cortex.

## Results

### MEMRI Studies

We verified that the order of Mn instillation (right nostril then left one or the reverse) did not affect Mn enhancement in the olfactory bulbs (ANOVA, F(1,6) = 0.09, p = 0.7746). In addition, whatever the instillation order, Mn enhancement in the right and left olfactory bulbs did not differ (ANOVA, F(1,6) = 2.412, p = 0.1714).

**Figure 1:**
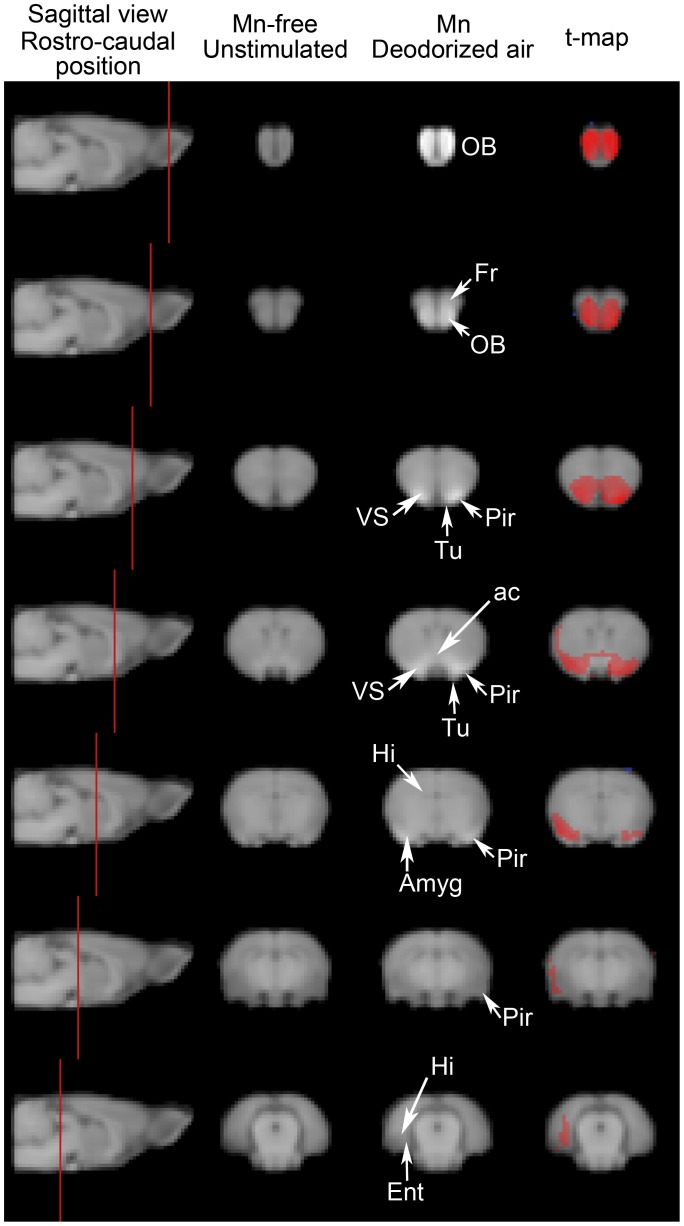
Figure **1. Mn accumulation along olfactory pathways in the absence of olfactory stimulation.** T1-weighted MEMRI images were obtained from the 9 rats that did not receive any Mn and were not submitted to any odorous stimulation and from the 7 rats that received 0.3 µmol Mn intranasally and were exposed to a continuous flow of deodorized air. The 16 MEMRI images were normalized both spatially and in intensity as described in the text. The mean intensity values of homologous voxels in the 2 groups were then compared using a Student’s two-tailed t-test. Left column: a parasagittal view of the rat brain intersected by vertical red lines indicating the position, along the rostro-caudal axis, of the coronal sections shown in the three other columns. Center columns: coronal sections through the T1-weighted normalized MEMRI images of the 9 Mn-free unstimulated rats (left) and the 7 Mn-injected rats exposed to deodorized air (right). Right column: statistical t-map overlaid on T1-weighted MEMRI image showing Mn enhancement. T-values in red represent voxels whose mean intensity in the Mn group was higher than that of their homologues in the Mn-free group (p<0.001). Abbreviations: ac anterior commissure, Amyg Amygdaloid nuclei, Ent entorhinal cortex, Fr frontal cortex, Hi hippocampus, OB olfactory bulb, Pir piriform cortex, Tu olfactory tubercle, VS ventral striatum.

Normalized T_1_-weighted images of Mn-free unstimulated rats and of Mn-injected rats exposed to deodorized air were compared on a voxel to voxel basis to yield a statistical t-map, which revealed contrast enhancement (i.e. Mn distribution) after 48 h of Mn progression. This is illustrated in Figure 1, which displays group-averaged images of coronal slices at 7 rostro-caudal levels and the resulting computed t-map, thresholded at p<0.001. Rostrally, the olfactory bulbs and the frontal cortex appeared labeled bilaterally. Mn loading encompassed structures anatomically close to the olfactory bulb, such as the anterior olfactory nucleus and the tenia tecta. At more caudal levels in the primary olfactory cortex, the anterior piriform cortex and the olfactory tubercle were also labeled bilaterally. Labeling also extended to the neighboring ventral striatum. The anterior commissure, through which the left and right primary olfactory cortices are connected, was also labeled. More caudally, labeling was predominant in the right side of the brain, in an area probably encompassing amygdaloid nuclei and the lateral dorsal part of the piriform cortex. Finally, in the caudal most part of the labeling, Mn accumulation was confined to the right side of the brain, in the inner side of the cortical mantle, probably in the entorhinal cortex and possibly also in the hippocampal area.

**Figure 2:**
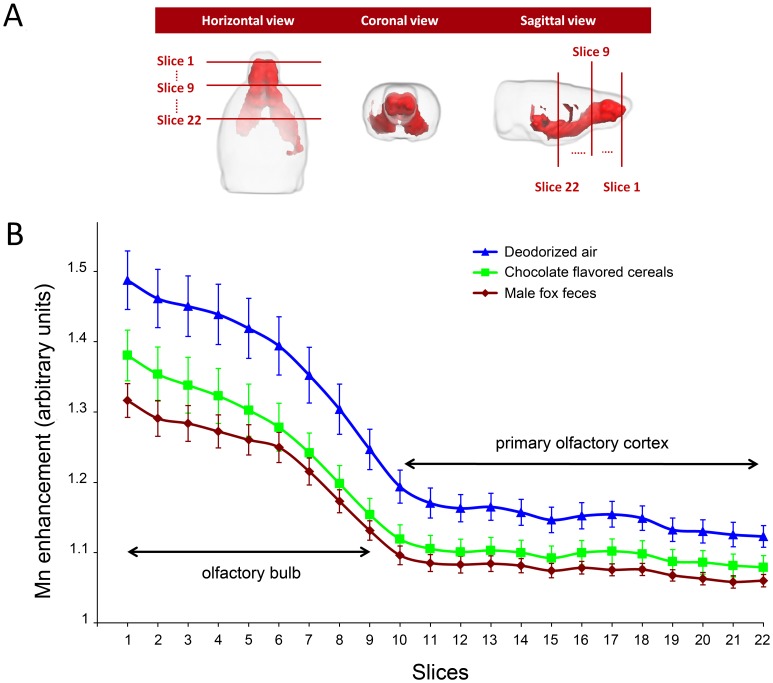
Effect of odorous stimulation on Mn enhancement along the olfactory pathways. (A) 3D views of the t-map shown in the right column of Figure 1. The intersection of this t-map and coronal planes at 22 rostro-caudal levels, (3 levels are shown: 1, 9 and 22) defined a region of interest (ROI) in which Mn enhancement was averaged to assess the effect of odorous stimulation. (B) Rostro-caudal distribution of the mean Mn enhancement in the 3 stimulated groups. At each level, the mean (± sem) Mn enhancement was calculated and compared using a one-way ANOVA. As indicated in the text, fox feces induced enhancement was always significantly different from deodorized air enhancement. Chocolate enhancement significantly differed from that of deodorized air only in slices 6 to 15 and in slice 17.

A 3D representation of the t-map (which represents the Mn load in the absence of any odorous stimulation) is displayed in Figure 2A. The volume of significant Mn enhancement (i.e. the Mn labeled volume) was used as a template to analyze the effect of odorous stimulation. This analysis was performed on bidimensional coronal ROIs defined as the intersections of the 3D t-map with 22 rostro-caudal coronal planes, 3 of which are shown in Figure 2A. Figure 2B shows, at each of these 22 levels, the mean value of T_1_-weighted image signal in these ROIs, for the deodorized air stimulated (control) group and the 2 odor-stimulated groups. In all groups, the mean T_1_-weighted image signal decreased markedly through the olfactory bulb (−15.5±1.3%, slices 1-9) and more moderately in the primary olfactory cortex and ventral striatal region (−4.3±1.4%, slices 10-22). A significant link was found between signal intensity and odorous stimulation at each of the 22 rostro-caudal levels (One way ANOVA 5.119≤ F(2,20) ≤7.226, 0.004≤p≤0.017). Post-hoc pairwise analysis (using the Holm-Sidak method) indicated that fox values were significantly lower than deodorized air ones (0.005≤p≤0.017) all along the 22 sections, while chocolate values differed from deodorized air ones (0.017≤p≤0.050) only in sections 6 to 17 (with the exception of section 16 in which p  = 0.052). Chocolate values were never statistically different from fox ones, although all along the rostro-caudal extent, chocolate contrast was consistently higher than fox contrast (see Figure 2B).

### MEMRI Contrast Enhancement and Fos Immunodetection in the Piriform Cortex

Following stimulation with male fox feces odor or chocolate flavored cereals, c-Fos immunoreactive neurons were detected in the anterior piriform cortex, and the results were compared with the Mn enhancement elicited by the same odors in ROIs encompassing the same brain region.

**Figure 3:**
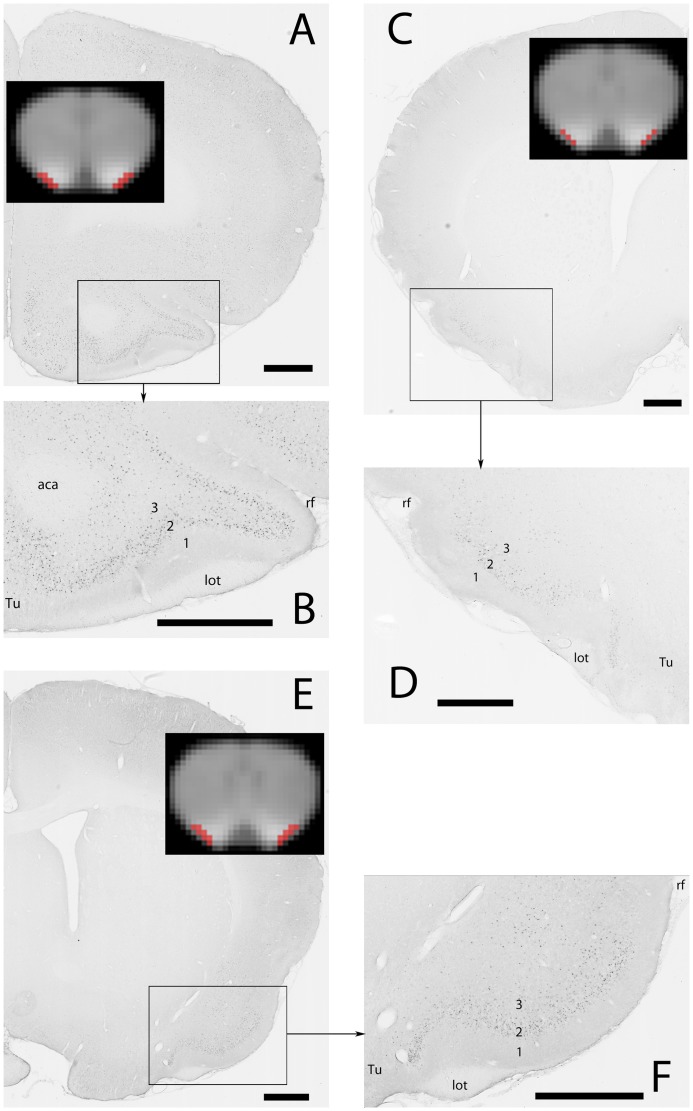
MEMRI contrast enhancement and c-Fos immunodetection in the anterior piriform cortex. A, C & E: photomicrographs of the right (C) and left (A, E) sides of the brain. MEMRI images of the corresponding slices are shown in inserts with the ROI encompassing the piriform cortex in red. B, D & F: enlargements of the regions delimited by the black rectangles in A, C & E, respectively. A, B: anterior region of the anterior piriform cortex; stimulation: chocolate flavored cereals C, D: medial region of the anterior piriform cortex; stimulation: empty container E, F: posterior region of the anterior piriform cortex; stimulation: fox feces Abbreviations (1), (2), (3): layers 1, 2 and 3 of the piriform cortex, (aca) anterior part of the anterior commissure, (lot) lateral olfactory tract, (rf) rhinal fissure, (Tu) olfactory tubercle. Scale bars (only for Fos images): 1 mm.

Whatever the odor, c-Fos^+^ neurons were observed in the anterior piriform cortex (Figure 3). Neurons were mostly located within layer 2 (Figure 3B, D and F), although a few could be found in layer 3. Almost no labeling was detected in layer 1. Along the medio-lateral axis, c-Fos^+^ neurons extended from the rhinal fissure laterally to the limit of the olfactory tubercle medially, which appeared much less labeled.

**Figure 4:**
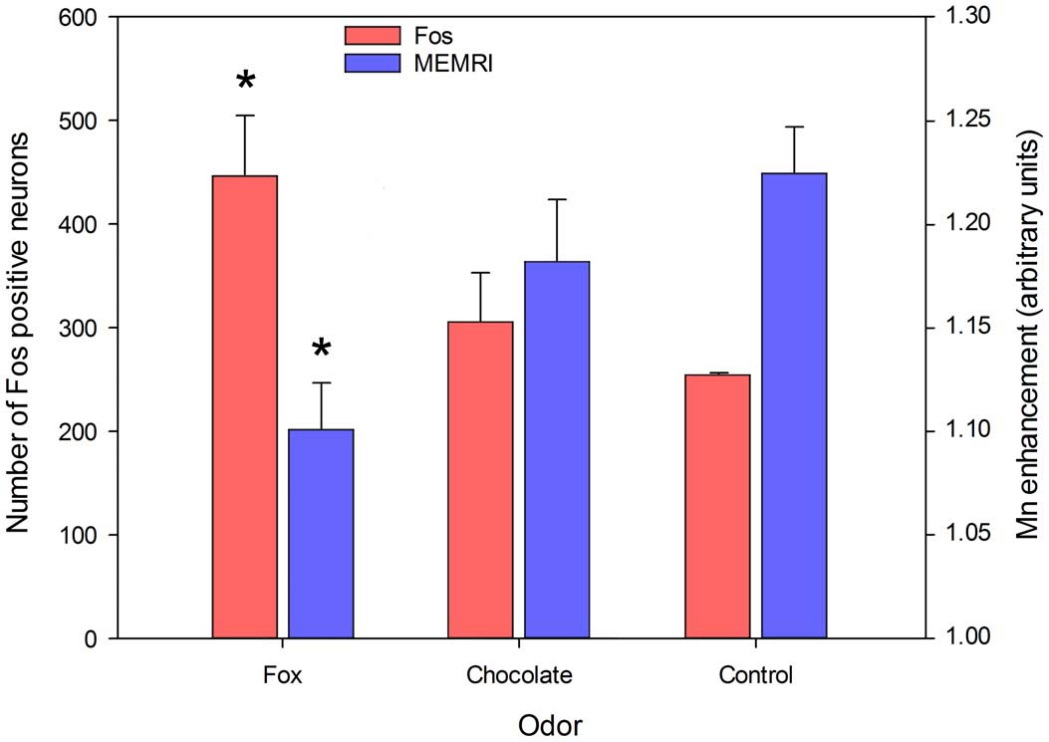
Comparison of c-Fos immunodetection and MEMRI signal. Red bars: number of c-Fos^+^ neurons within the anterior piriform cortex of rats stimulated by the odors released by a receptacle either empty or containing chocolate flavored cereals or fox feces. Means ± s.e.m. are shown. *: p<0.05 when compared to the corresponding empty container (control) value. y-axis legend is on the left. Blue bars: Mn contrast enhancement within the anterior piriform cortex in ROIs homologous to the regions examined for Fos immunoreactivity (as shown in Figure 3). Odorous stimulation was an airflow odorized with either male fox feces or chocolate flavored cereals. Means ± s.e.m. are shown. *: p<0.05 when compared to the corresponding deodorized airflow (control) value. y-axis legend is on the right.

Three rostro-caudal levels were analyzed in the anterior piriform cortex. The rostral most one was located where the olfactory bulb is no longer separated from the orbital cortex (Plate 11, Bregma 3 mm in the Paxinos and Watson rat brain atlas [41]), the posterior one at the closure of the anterior commissure (Plate 34, Bregma −0,12 mm), which is just rostral to the end of the lateral olfactory tract, the landmark defining the caudal end of the anterior piriform cortex [7] and the central one approximately in the middle of this extension. For each animal and each level, c-Fos^+^ neurons were numbered separately in the right and left piriform cortices. Since in no case was a difference detected between sides (Student’s t-test in all comparisons, p>0.05), neuron numbers were averaged to provide a unique value per level and per rat. Finally, in fox feces and chocolate odor stimulated rats, the number of c-Fos^+^ neurons did not change at the 3 rostro-caudal levels examined. Therefore, the 3 values were averaged to give one number of c-Fos^+^ neurons per odor and per rat. Results are shown in Figure 4. They indicate that the number of activated neurons significantly depended upon the stimulating odor (one way ANOVA, F(2,6) = 2.531, p = 0.048) and post-hoc pairwise analysis revealed that this resulted from stimulation with fox feces odor, which activated a greater number of neurons than the empty container (p = 0.041, Holm-Sidak method). Chocolate odor remained without any statistically significant effect (p = 0.436, Holm-Sidak method).

Fos labeling was compared to the MEMRI signal in the anterior piriform cortex. Within the Mn-enhanced 3D volume, three virtual slices were chosen that matched the piriform cortex levels analyzed for c-Fos immunoreactivity (inserts in Figure 3A, C and E). These slices corresponded to levels 16, 17 and 18 on Figure 2B. ROIs encompassing the piriform cortex were manually delineated and the MEMRI signal was determined. Results are shown in Figure 4. A significant relationship was found between the MEMRI signal and the stimulating odor (one way ANOVA, F(2,20) = 6.007, p = 0.009). Pairwise post-hoc analysis indicated that this was due to the effect of fox feces stimulation that gave a signal significantly lower (p = 0.009, Holm-Sidak method) than that given by deodorized air. Similarly to what was found with c-Fos activation, chocolate odor MEMRI signal did not differ from deodorized air signal.

## Discussion

In the present study, we show that two different natural odors affect differently the MEMRI signal, suggesting that MEMRI, as an *in vivo* imaging technique, can reveal differential activation of neuronal pathways. This validates MEMRI as a functional MRI approach to explore deep olfactory brain regions.

Mn, injected intranasally, has already been used as a tracer of olfactory pathways [42]–[44] and has also been used for functional analyses, although limited to the olfactory bulb [33], [45]. However, as we have shown recently [37], doses used for anatomical purposes cannot be employed in functional studies, because of Mn neurotoxicity [36]. The dose of 0.3 µmol, used in the present experiments ensured both preservation of olfactory capacities, penetration of Mn into olfactory pathways and its detection up to the entorhinal cortex. It may be noted that Mn distribution in distal most brain regions was somewhat asymmetric, with a heavier labeling in the right side of the brain. The mode of calculation of the mean Mn load, combined to the low concentration of Mn in distal regions may account for this observation. It should be noted, however, that our analysis was limited to a rostro-caudal extension in which Mn load was bilateral (i.e. olfactory bulbs, anterior olfactory nucleus, lateral olfactoty tract, piriform cortex and olfactory tubercule).

In the absence of odorous stimulation (i.e. only a flow of deodorized air), the MEMRI signal diminished along the rostro-caudal axis. The decrease was biphasic: rapid within the olfactory bulb, slower thereafter, with an inflexion at the caudal end of the olfactory bulb. One possible explanation is that, within the olfactory bulb, the concentration of Mn at a given coronal plane directly depends on the number of axons of olfactory receptor neurons (ORN) crossing this plane to innervate olfactory glomeruli at this position or further. Because glomeruli are organized as a single layer envelope in the external part of the olfactory bulb, the number of ORN axons rapidly decreases with the rostro-caudal position, possibly explaining the rapid decrease of Mn enhancement. Caudal to the olfactory bulb, there is no longer any ORN axon. The only neurons containing Mn are at least one synapse away from the glomerular layer. The efficiency of synapse crossing by Mn may account for the inflexion of the Mn enhancement curve. Further on, mitral and tufted cells innervate a number of structures with a large and diffuse distribution of their projections [46], probably accounting for a slower decrease of Mn enhancement in the primary olfactory cortex.

Odorous stimulation with fox feces or chocolate flavored cereals also elicited biphasic MEMRI profiles along the rostro-caudal axis. In addition, signal intensity was linked to odor. Indeed, when compared to deodorized air, fox feces odor elicited a significantly lower signal all along the rostro-caudal extension of the Mn loaded volume while the effect of chocolate odor was restricted to the medial part of the Mn loaded volume. Also, in manually delineated ROIs encompassing the anterior piriform cortex (inserts in Figure 3 corresponding to levels 16–18 of Figure 2B), the MEMRI signal elicited by fox feces odor was significantly lower than that elicited by deodorized air, while chocolate signal was not. These results may be interpreted in light of c-Fos immunodetection. In the anterior piriform cortex, the number of neurons activated by fox feces odor was significantly higher than that of neurons activated by an empty container, whereas the number of neurons activated by chocolate odor was not. Given that c-Fos activation is proportional to stimulus intensity [47], one may conclude that fox feces odor is a more potent stimulus than chocolate odor. This also implies that the stronger the odorous stimulus, the lower the MEMRI signal.

The conclusion that fox feces odor is a more powerful stimulus than a food odor is in keeping with a recent report [48] showing a greater electrophysiological response of olfactory tubercle neurons to fox feces odor than to food odor and to a control odor. It is also in agreement with behavioral data showing that emotional behavior in rats is greater when induced by fox feces odor than by conspecific feces odor [39]. Altogether, electrophysiological, behavioral, MEMRI and c-Fos results show that a predator odor represents a more salient stimulus than other odors and is differently processed in the primary olfactory cortex.

The conclusion that the MEMRI signal is inversely proportional to stimulus intensity is in keeping with recent results obtained in the olfactory bulb of the starling [35]. In these birds, the MEMRI signal elicited by milfoil odor is lower in spring, when milfoil odor carries an additional message linked to reproduction. The question remains of the mechanism responsible for a lower MEMRI signal in response to a more salient stimulus. While it is accepted that Mn uptake is proportional to neuronal activation [49], it is also known that a higher neuronal activation elicits a faster axonal transport [32] and consequently a higher synaptic release of Mn. Since MEMRI detection of extracellular Mn is not as efficient as that of intracellular Mn [50], a greater stimulation may well lead to a lower MEMRI signal, MEMRI contrast reflecting the intraneuronal Mn content left after stimulation. This may be particularly true under our experimental conditions of a limited supply of extracellular Mn and a prolonged stimulation times, which limit the entry of Mn into neurons and give time to neurons to release Mn. Alternatively, a lower MEMRI signal may result from a lower Mn uptake, as the message is more salient, due to activity-dependent lateral inhibitory mechanisms in the olfactory bulb [51]. Such mechanisms, which enhance the salience of a biologically relevant odor over other olfactory signals (for review, see [52]), may also well exist in the primary olfactory cortex, as suggested by its anatomical organization (for review, see [53]). However, this hypothesis does not fit with our present observation of a higher c-Fos signal in the piriform cortex and the report of a higher number of activated neurons in the olfactory tubercule of rats stimulated by fox feces odor [48]. In any case, it is worth noting that differences of MEMRI signal exist in the first slice of the Mn loaded volume, which is the rostral most part of the olfactory bulb. This suggests that whatever the mechanism that links Mn uptake/transport to intensity of the odorous signal, it is likely triggered before ORN axons reach the olfactory bulb, i.e. in the olfactory epithelium.

Finally, the factors that make fox feces odor a more salient stimulus than chocolate odor remain unclear. One may invoke the intensity of the applied stimuli, or the complexity of the odorous bouquets, or the biological significance of these odors (i.e. predator vs food). Our stimulation protocols, either for MEMRI or for c-Fos immunodetection, did not include any device to control for vapor pressure or intensity of the stimulus. Both fox feces and chocolate flavored cereals contain hundreds of molecular odorants in varying concentrations. In view of the widespread distribution of the projections of mitral and tufted cells connected to a single glomerulus [46] and of the apparent lack of spatial organization within the piriform cortex [11], it seems unlikely that bouquets containing hundreds of molecular odorants may be discriminated by their degree of recruitment of the primary olfactory cortex. With respect to concentration/intensity of the odors, although we have applied them in a reproducible manner, in MEMRI as well as in c-Fos experiments, we have no indication regarding their perceived intensity by the animals. It is all the more difficult to evaluate intensity that we were dealing with two different odors. Experiments by De Groof and coworkers [35] are very interesting in this respect. The observation that the same milfoil odor may generate a lower MEMRI signal in spring, when this odor conveys an additional significance linked to reproduction, strongly suggest that MEMRI signal, at least in the case of biologically relevant bouquets, relates to the significance of the odorous signal and not to its composition. This hypothesis is in keeping with recent results of our lab [48] showing that the number of activated neurons in the olfactory tubercle of male rats varies with the odorous stimulus, ranked by decreasing efficacy as follows: predator odor, conspecific sexually receptive female odor, food odor, control odor.

In conclusion, MEMRI appears to be a promising technique for the functional imaging of olfactory processes in deep brain regions, under conditions close to natural perception in awake animals. We were able to unmask functional differences in the processing of biologically relevant odors. These results were obtained following Mn instillation in the nostrils followed by imaging 48 hours later, time needed for Mn to reach deep olfactory brain structures. A possible improvement might be to search for a better route of Mn administration in order to ensure a more homogeneous and quicker brain bioavailability. This would allow shortening the delay between Mn administration and imaging, as well as reducing neurotoxic effects of Mn. Together with the use of a higher magnetic field, this might ensure a better functional and spatial discrimination between the various structures that compose the primary olfactory cortex and a better discrimination between odor effects.

## Materials and Methods

The study was in full compliance with the guidelines of the European community (EUVD 86/609/EEC) for the care and use of the laboratory animals.

### Animals

Forty-nine adult male Brown-Norway rats weighing 234±18 g (René Janvier, Le Genest-Saint-Isle, France) were housed individually under a reverse 12 h:12 h light - dark cycle (light on at 20:00). Forty rats were used for functional imaging experiments:

- 9 rats did not receive any Mn nor olfactory stimulation and were used for the normalization process
- 8 rats received Mn in the right nostril then in the left one (4 rats) or in the reverse order (4 rats) and were stimulated by a deodorized airflow to assess for a possible effect of the order of instillation
- 7 rats received Mn in both nostrils (in a random order) before being stimulated by a deodorized airflow (control for the experiments on odor effect).
- 16 rats received Mn in both nostrils (in a random order) before being stimulated by an airflow odorized with male fox feces (8 rats) or chocolate flavored cereals (8 rats).

Another group of 9 rats was used in c-Fos experiments. These rats were submitted to olfactory stimulation (3 rats per group) using either male fox feces or chocolate flavored cereals or an empty container. These 9 rats plus the 23 rats of the 2 groups that received Mn in a random order were fasted during 48 hours before experiments.

### MEMRI Studies

The 9 rats that did not receive Mn or odorous stimulation just underwent anesthesia. Imaging was performed 48 hours later.

The second group (8 rats) was imaged 4 hours after Mn administration.

The remaining 23 rats that underwent odorous stimulation were fed, nine days before the experiment, with 20 g/day of standard rodent food (M25 diet, Special Diets Services, Witham, UK), except those stimulated with chocolate odor, which were fed with a mixture of rodent diet (14 g) and chocolate flavored cereals (6 g, Chocapic™, Nestlé, Switzerland). These rats were imaged 48 hours after Mn administration.

#### Manganese administration

Rats were sedated for 5 min with 4% isoflurane and placed in the supine position. Three microliters of a 100 mM MnCl_2_-4H_2_O solution (Sigma-Aldrich, France) were injected into both nostrils through a polyethylene catheter (PE 10 tubing) attached to a Hamilton syringe. Upon wakening (approximately 1-2 min after Mn administration), rats were individually placed into a clean cage with free access to water only.

#### Odorous stimulation

Upon return into their cage, rats were submitted during 48 hours to a constant air flow of 1.5 L/min delivered by means of a custom made device connected to the cage by a sniffing port. Air, deodorized by means of a charcoal filter, passed through either an empty bottle or a bottle containing an odorous sample before being pulsed into the animal cage. The odorous sample (renewed every 12 hours) consisted in either 100 g of crushed chocolate flavored cereals or 50 g of male fox feces, kindly provided by Dr Franck Boué (ANSES, Malzeville, France). Air was blown through the bottle containing the odorous sample during 5 min every half hour, and through the empty bottle otherwise.

#### Image acquisition

At the end of the stimulation period, rats were anaesthetized under urethane and imaged. Urethane was chosen because it provides long periods of anaesthesia with low respiratory depression and it avoids inhalation of an odorous anesthetic.

Images were acquired on a Bruker (Bruker GmbH, Ettlingen, Germany) 4.7 T/40 cm horizontal magnet equipped with a 12-cm gradient coil and interfaced to an Avance console. A 9-cm-diameter linearly-polarized birdcage resonator was used for emission and reception. Animals were placed in a rat-tailored bed equipped with a three point-fixation system (tooth-bar and ear-plugs) and a temperature-controlled warming blanket. Physiological conditions were monitored using breathing and temperature probes. T1-weighted images covering the whole brain were acquired by 3D rapid acquisition with a relaxation enhancement (RARE) sequence. Centric encoding was used to keep echo time short ( = 5.5 ms). The other scan parameters were: TR  = 500 ms, matrix  = 60^3^, isotropic voxel size  =  (500 µm)^3^, flip angle –90°, 4 echoes per TR, acquisition time  = 7 min 30 s. Acquisitions were repeated for 150 min, yielding 20 images per rat. Each series was first motion-corrected via rigid registration using automated image registration [54]) and then averaged.

#### Image processing and analysis

Images were successively segmented, normalized iteratively in both space and intensity [38] and smoothed using 3D Gaussian filter (FWHM  = 0.5 mm) in order to take into account the known error of interindividual registration. The reference signal for intensity normalization was measured in the tongue.

A possible effect of the order of Mn instillation on signal enhancement was assessed by a two-way mixed-model ANOVA performed on MEMRI signal averaged over the right and left olfactory bulbs. The instillation order was used as between subjects factor and olfactory bulb side (left or right) as within and between subjects factor.

Spatial distribution of Mn accumulation was determined by comparing, voxelwise and using a two-tailed student’s t-test, images of the Mn-free unstimulated rats to the images of the Mn-injected, deodorized air-stimulated rats. The resulting t-map was thresholded at p<0.001 to reveal the 3D volume of Mn-enhanced regions. Analysis of the effects of olfactory stimulation was performed by comparing signal intensities measured in this 3D volume for rats stimulated either with deodorized air or chocolate odorized air or fox feces odorized air. In this purpose, signal intensity was averaged over the intersections between 22 contiguous 500 µm-thick coronal planes and this 3D volume along the rostro-caudal axis. At each level, average intensities corresponding to all 3 odors were compared by a one-way ANOVA followed by post-hoc pairwise comparison using the Holm-Sidak method.

In order to compare MEMRI results with those of c-Fos immunodetection, anatomical ROIs corresponding to the same rostro-caudal levels in the piriform cortex were manually delineated, taking as a reference the Rat Brain Atlas of Paxinos and Watson [41]. Comparisons were performed using one-way ANOVA followed by post-hoc pairwise comparison using the Holm-Sidak method.

### c-Fos immunodetection

#### Odorous stimulation

On the day of experiment, the rat (fasted during the past 48 hours) was placed during 5 min for habituation in a glass stimulation arena (LxWxH: 50×30×30 cm^3^) with clean sawdust as litter. Olfactory stimulation consisted in presenting during 30 min an aluminum foil either empty or supporting a piece of chocolate cereal or a sample of male fox feces. This aluminum foil was put in a receptacle covered with a bolted-on perforated lid. Three rats were successively stimulated on the same day (each with a different odorous sample). Between two experiments, the aluminum foil and the litter were discarded, the arena was thoroughly cleaned and the stimulation room ventilated for 20 min.

#### Tissue preparation

At the end of the session, rats were returned to their home cage and deeply anaesthetized 60 min later by an i.p. injection of 1 mL of a 60 mg/mL solution of sodium pentobarbital (CEVA Santé Animale, Libourne, France). They were intracardially perfused with 200 mL Ringer lactate solution (CDM, Lavoisier, France) at 4°C followed by 500 mL 10% neutral buffered formalin (Diapath, Microm Microtech, France), pH 7.2–7.4 at 4°C. Brains were dissected out, postfixed overnight in the second perfusion solution and dipped during 24 h in a 15% sucrose solution and during 48 h in a 30% sucrose solution for cryoprotection.

Brains were frozen and sampled using a cryostat (Leica 3050S) in 40 µm thick coronal sections from the olfactory bulbs to the closing of the anterior commissure. Two consecutive sections were collected every 4 sections. One was used for Nissl staining and the following one for c-Fos immunohistochemistry.

#### Immunohistochemistry

Slices were dried at room temperature, rinsed 2×10 min in phosphate buffered saline (PBS, Sigma-Aldrich, France) and preincubated during 45 min in a moist chamber with 500 µL/slide of 0.25% (v/v) Triton X-100 (Sigma-Aldrich, France) in PBS containing 1.5% (v/v) goat serum (Vector Labs, AbCys, France). Slices were incubated overnight at room temperature with a polyclonal rabbit anti-rat c-Fos antibody (Calbiochem, VWR, France) at a dilution of 1/20000 in PBS-Triton containing 1.5% goat serum (v/v). They were rinsed 3 times in PBS containing 0.1% skimmed milk and incubated during 90 min with biotinylated anti-rabbit antibody (Vector Labs, AbCys, France) diluted 1/400 in PBS-Triton containing 0.1% bovine serum albumin. Slices were rinsed 3×10 min with PBS, dipped during 10 min in PBS containing 0.3% H_2_O_2_ and rinsed 3×10 min in PBS. They were incubated with the Avidin-Biotin complex (Vectastain, Vector Labs, AbCys, France) during 45 min and rinsed 2×10 minutes in PBS and during 10 min in 50 mM Tris buffer (pH 7.6). Labeling was revealed using nickel-enhanced diamino-benzidine (SK-4100, Vector Labs, AbCys, France).

#### Image digitization and analysis

Section images were digitized with an automatic image digitizer (Nanozoomer, Hamamatsu, France). The piriform cortex was delineated according to Paxinos and Watson (2007). c-Fos immunoreactive (c-Fos+) neurons were identified and numbered using Fiji software (http://pacific.mpi-cbg.de/wiki/index.php/Fiji). Effect of odor on c-Fos activation was searched for using a one-way ANOVA. They were performed using SigmaPlot 12 (Systat, Chicago, IL, USA). Normality of distributions was checked using a Shapiro-Wilk test and equality of variances using a Levene median test. Since, in the present study, both conditions were always met, direct pairwise comparisons were done using t-tests and multiple group comparisons one-way ANOVAs followed by Holm-Sidak pairwise post-hoc comparisons. Differences were considered significant for p<0.05.
